# Provision of hearing technology in children and adolescents with permanent hearing loss in Germany

**DOI:** 10.1007/s00106-025-01617-0

**Published:** 2025-06-17

**Authors:** Elena Pützer, Heike van de Sand, Jasmin Filip, Ingrid Schubert, Ursula Marschall, Ingo Meyer, Karolin Schäfer

**Affiliations:** 1 Faculty of Human Sciences, Department of Special Education and Rehabilitation, Education and Aural Rehabilitation of People who are Deaf or Hard of Hearing, University of Cologne, Klosterstr. 79b, 50931 Cologne, Germany; 2 PMV Research Group, Medical Faculty and University Hospital Cologne, University of Cologne, Cologne, Germany; 3 Department Medicine/Health Care Research, Barmer, Wuppertal, Germany; 4 Institute for Special Needs Education (d/Deaf and Hard of Hearing), University of Duisburg-Essen, Essen, Germany

**Keywords:** Deaf/Hard of hearing, Hearing devices, Early detection, Health service research, Claims data, Hörschädigung, Hörhilfen, Früherkennung, Versorgungsforschung, Routinedaten

## Abstract

**Background:**

To date, data on the prevalence and age at first management of permanent childhood hearing loss in Germany are lacking.

**Objective:**

This study aims to depict how often and at what age children and adolescents receive their (first) hearing technology.

**Materials and methods:**

In this study, we analyzed claims data from a large German statutory health insurance company (BARMER). A cross-sectional study determined the provision of hearing devices and cochlear implants for children and adolescents aged under 18 years with permanent hearing loss from 2010 to 2020. A longitudinal analysis of a cohort of children born in 2010 was performed to gain insights about age at first management with hearing technology during the first 10 years of life.

**Results:**

Between 2010 and 2020, approximately 2800 to 3600 children and adolescents per year were provided with hearing devices and 10 to 30 with cochlear implants. In the 2010 birth cohort, 1.22% of children received their first prescription for hearing devices before the age of 10. The proportionately highest number of first prescriptions was found between 3 and 6 years. In 2020, particularly few children gained access to hearing technology.

**Conclusion:**

The analysis of the prescribed hearing systems reveals inaccuracies in documentation but also a possible care gap in hearing loss management. For a large percentage of children and adolescents, management of hearing loss took place after the age of 1. The frequent initial provision of hearing technology at preschool age indicates that the proportion of hearing loss that is acquired, detected late, or treated late remained quite high even after the introduction of newborn hearing screening. There is an obvious need for comprehensive tracking of children who fail newborn hearing screening and for other screening and hearing tests. The data for 2020 suggest that hearing loss was diagnosed and treated later due to the COVID-19 pandemic.

## Abstract (de)

**Hintergrund:**

Bisher liegen keine Daten zu Versorgungshäufigkeiten und -zeitpunkten von Kindern und Jugendlichen mit permanenter Hörstörung in Deutschland vor.

**Ziel der Arbeit:**

Ziel der Studie ist es, herauszufinden, wie häufig und in welchem Alter Kinder (erstmalig) ein technisches Hörsystem erhielten.

**Material und Methoden:**

Mithilfe von Routinedaten einer der größten deutschen gesetzlichen Krankenkassen (BARMER) wurde eine Querschnittsanalyse für die Jahre 2010 bis 2020 vorgenommen, um zu ermitteln, wie viele Kinder und Jugendliche mit permanenter Hörstörung unter 18 Jahren mit Hörhilfen nach Definition der Hilfsmittelrichtlinie und Cochleaimplantaten versorgt wurden. In einer Längsschnittanalyse einer Kohorte von Kindern des Geburtsjahrgangs 2010 wurde zusätzlich das Alter bei der Verordnung der ersten technischen Hörsysteme in den ersten zehn Lebensjahren betrachtet.

**Ergebnisse:**

Zwischen 2010 und 2020 erhielten jährlich ca. 2800 bis 3600 Kinder und Jugendliche eine Hörhilfe und 10 bis 30 ein Cochleaimplantat. In der Geburtskohorte 2010 erhielten 1,22 % der Kinder in ihren ersten 10 Lebensjahren eine Hörhilfe. Darunter waren die meisten Erstverordnungen im Alter zwischen 3 und 6 Jahren zu finden. Im Jahr 2020 wurden besonders wenige Kinder mit technischen Hörsystemen versorgt.

**Schlussfolgerung:**

Bei der Analyse der verordneten Hörsysteme wurden Ungenauigkeiten in der Dokumentation, aber auch eine mögliche Versorgungslücke sichtbar. Die häufige Erstverordnung von Hörsystemen nach dem ersten Lebensjahr – insbesondere im Kindergartenalter – deutet an, dass der Anteil erworbener, später erkannter oder später versorgter Hörstörungen auch nach Einführung des Neugeborenen-Hörscreenings durchaus hoch war. Es wird ein Bedarf an umfassenden Trackings von auffälligen Kindern im Rahmen des Neugeborenen-Hörscreenings sowie weiterer Erfassungsmethoden offensichtlich. Die Daten für das Jahr 2020 lassen vermuten, dass Hörstörungen aufgrund der COVID-19-Pandemie später diagnostiziert sowie versorgt wurden.

Early treatment of peripheral hearing loss in children and adolescents is of great importance in view of the effects of untreated hearing loss on various areas of development. Data on the provision of hearing technology can be used to map the state of hearing care and optimize processes on this basis.

## Background

With the introduction of universal newborn hearing screening (UNHS) in Germany in 2009, an important foundation stone was laid to achieve the goal of early detection and intervention of children with peripheral hearing loss [1]. This objective is based on study results that show that children with early provision of hearing technology and early intervention have advantages over children with late provision in various areas of development—especially language development [7, 9, 17]. However, no comprehensive data are yet available on the age at which affected children are provided with hearing technology for the first time.

The prevalence of bilateral, permanent, congenital hearing loss in children in Germany is estimated at 0.13% based on data from the UNHS in 2011/2012 [15]. Data on the prevalence of hearing loss throughout childhood and adolescence can only be found in international studies and vary considerably—between 0.1% and 17.9%. This is partly due to different data collection methods and inclusion criteria [26]. In a pilot study with Germany-wide claims data of statutory health insurance providers, the administrative prevalence of hearing loss for children under 18 years was estimated at 0.61–0.69% for the period 2010–2019 [23]. An extrapolation to the German population of 2019 [4] suggests that a total of 83,012 children and adolescents in Germany were affected by hearing loss in 2019 [23].

An analysis of claims data offers the possibility to map the state of hearing care and management for children with hearing loss in the population. The present study, which also included the estimation of administrative prevalence [23], uses routine data to retrospectively collect information on the onset of childhood hearing loss management.

## Methods

In the present study, claims data from statutory health insurance providers were analyzed. These large datasets are collected and stored by health insurance providers for accounting purposes and can be used for research under certain conditions [13, 18]. For this study, claims data from the health insurance company BARMER were evaluated, covering approximately 9 million insured individuals nationwide (as of 2020). The data were provided by BARMER in a virtual computing environment separate from BARMER’s data inventory, with exclusively anonymized data, ensuring compliance with data protection regulations [21]. Researchers can access these data upon request. In addition to basic demographic data about insured individuals, information on the utilization of services from various sectors is available. In this project, we used diagnoses coded with ICD-10 from both outpatient and inpatient sectors; codes from the German classification for the encoding of operations, procedures, and general medical measures (operation and procedure codes, *Operationen- und Prozedurenschlüssel*, OPS); and prescriptions for medical aids, which can be combined using a pseudonymized insurance number (for details on claims data, see [22]).

First, we developed a case definition to include all children with permanent hearing loss. To confirm the documented ICD-10 diagnosis for internal validation purposes [19], it was assumed that hearing loss was present if either a diagnosis of hearing loss due to conductive or sensorineural disorders (ICD-10: H90.–) was coded (as the inpatient main diagnosis or as a confirmed outpatient diagnosis in at least two of eight quarters) or if hearing technology was prescribed. During the development of the case definition, we also evaluated ICD code H91.–. It was found that this code—and primarily code H91.9—was documented much more frequently than code H90.–, leading to implausibly high case numbers. For children who received hearing devices, we predominantly found internally validated H90.– diagnosis. Finally, for reasons of plausibility, only the H90.– codes were included.

Hearing devices were identified using their item number in the register of approved aids and appliances used by the German statutory health insurance funds (German medical aids index, *GKV-Hilfsmittelverzeichnis*; [8]). All devices in the product group of “hearing devices” (with “13” as the first digits of the item number) were included. This allowed us to capture devices such as hearing aids, bone conduction systems, or hearing assistive technology systems. The hearing devices can be identified as specific hearing aids or hearing assistive technology systems if the billed numbers are listed in the medical aids index. Since cochlear implants are classified as prosthetics and are not listed in the medical aids index, they were identified using the German operation and procedure codes (OPS-Version 2021), i.e., code “5-209.2: Insertion of a cochlear implant” [3].

Based on these data, we conducted a cross-sectional analysis for each year between 2010 (*N* = 1,254,296) and 2020 (*N* = 1,174,507). The aim was to determine the percentage of continuously insured children and adolescents with peripheral hearing loss who received at least one prescription for a hearing device or had cochlear implant surgery by the age of 18. In the cross-sectional analysis, each year was analyzed individually. This means prescriptions of hearing devices or cochlear implant surgeries are evaluated for cases from that respective year. In a longitudinal analysis of a cohort of children born in 2010 (*N* = 38,705), we determined the percentage and age of children who were provided with hearing technology within the first 10 years of life.

## Results

Table 1 provides an overview of children under the age of 18 with hearing loss with at least one documented prescription for a hearing device or cochlear implant surgery per year. The term “children with hearing loss” refers to all cases under 18 years of age where the diagnosis was documented according to the specified case definition in the respective year, standardized to the German population. Between 2010 and 2020, the number of children with hearing loss ranged from 4874 to 8640. For these children, we evaluated prescriptions for hearing devices and cochlear implantations per year. Children who were already provided with devices were only recorded in subsequent years if another prescription or implantation was documented (e.g., prescriptions for new devices or for the contralateral ear). If children received both a hearing device and a cochlear implant in the same year, they are listed in both categories. In the years we evaluated, 2799–3592 children received a prescription for hearing devices per year. Between 13 and 32 children per year had cochlear implant surgery.

**Table 1:** Interventions with hearing technology for children and adolescents with hearing loss

Year	2010	2011	2012	2013	2014	2015	2016	2017	2018	2019	2020
*Children with hearing loss*	8640	8289	8137	8076	8003	7820	7359	8197	7761	7289	4874
*Hearing devices*	3511	3592	3571	3533	3386	3179	3131	3449	3388	3400	2799
Percentage	40.6	43.3	43.9	43.7	42.3	40.7	42.6	42.1	43.7	46.7	57.4
*Cochlear implants*	30	13	23	17	18	32	17	17	22	21	28
Percentage	0.3	0.2	0.3	0.2	0.2	0.4	0.2	0.2	0.3	0.3	0.6

The data refer to all children insured by BARMER who meet the criteria of the case definition “permanent hearing loss,” standardized to the German population for each respective year. “Hearing devices” refer to devices with an item number in the German medical aids index with “13” as the first two digits. Children with cochlear implant surgery and prescriptions for hearing device in the same year are listed in both categories

The item number in the German medical aids index usually allows the identification of a specific hearing device. However, we found several item numbers that are not allocated in the medical aids index. Therefore, the product group “hearing devices” was presented as a whole. Looking at different categories of product groups, between 2010 and 2019, the percentage of hearing aids ranged from 31.6% to 39.4% (mean: 36.1%), hearing assistive technology systems from 4.7% to 7.7% (mean: 6.1%), and unspecified hearing devices from 54.5% to 60.7% (mean: 57.8%). For example, in 2019 out of a total of 4399 prescriptions for hearing devices with “13” as the first two digits, approximately 31.6% could be identified as hearing aids and 7.7% as hearing assistive technology systems. Overall, 60.7% could not be assigned to either of the two groups due to their coding.

In 2020, the percentage of interventions with hearing technology deviated upward compared to previous years. However, the absolute numbers remained constant for cochlear implant surgeries. Absolute numbers of prescriptions for hearing devices were even lower than in previous years. The higher percentage of interventions with hearing technology is due to a decrease in the population of children and adolescents with diagnosed hearing loss.

### Age at first prescription of hearing devices

**Fig. 1:**
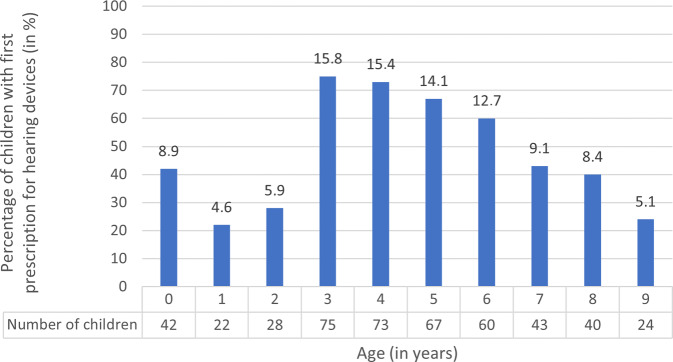
Distribution of first prescriptions for hearing devices by age (*n* = 474, children of the 2010 birth cohort with hearing devices)

For the birth cohort of 2010 (*N* = 38,705), we analyzed the time of the first prescription for hearing devices. A prescription for hearing devices with item number 13 in the medical aids index was documented for 1.22% of the cohort (474 children), within the first 10 years of life. We determined the frequency distribution of first prescriptions by age for this group (Fig. 1). Most first prescriptions for hearing devices were issued between the ages of 3 and 6 years. The smallest percentages were found among children aged between 1 and 2 years and also 9 years. Overall, more boys than girls received hearing devices (269 boys vs. 205 girls). Within the first 10 years of life, 14 of the 474 children received a cochlear implant. The first cochlear implant was implanted within the first 4 years of life for the majority of children (10 out of 14).

## Discussion

### Age at intervention with hearing devices

Previous studies reported the prevalence of congenital hearing loss in children to be between 0.13% and 0.16% [2, 10, 15, 24]. For children up to 18 years of age, a higher prevalence of 0.69% was observed, indicating that many children receive the diagnosis of hearing loss later [23]. This is reflected in the present study, as the majority of children and adolescents from the 2010 birth cohort received hearing devices after their first year of life. Only 8.9% of all children with hearing devices received the device in their first year of life. Late hearing loss management may occur either because the hearing loss was acquired or was initially undetected.

Most often, the first prescription for hearing devices was documented between the ages of 3 and 6 years, with 12.7 to 15.8%, respectively. This age group accounts for a total of 58% of all children in the cohort who received hearing devices within the first 10 years of life. The frequent first prescriptions of hearing devices and higher prevalence rates during preschool age indicate that the proportion of acquired or undiagnosed hearing loss in the 2010 birth cohort was substantial. It is possible that some children with late hearing loss management were not screened through UNHS or that a refer result was lost to follow-up. Evaluations of the UNHS have already shown that tracking children with refer results from initial screenings is challenging [14, 15]. Overall, only for 54% of all children who failed the initial screening in 2018 was a final result documented [14]. The range of cases without a final diagnosis in the screening centers of different federal states in Germany varies from 9% to 100%. Therefore, it remains an important task to follow up on children with refer results in the UNHS until the findings are conclusive so as to enable early intervention.

However, even if a child passed UNHS, childhood hearing loss cannot be ruled out [12]. An evaluation of the German UNHS found that children who had passed UNHS received their final diagnosis very late [15]. Data from the present study show that the percentage of prescriptions for hearing devices in children aged 1 and 2 years is particularly low (4.6% and 5.9% of all children with hearing devices, respectively). A reason for this could be a false sense of security because the UNHS was passed [12]. Additionally, at this age, there are fewer signs of developmental delay due to the early stage of language development. Therefore, it is important to inform parents of children who passed UNHS that the occurrence of hearing loss at a later age cannot be ruled out.

Based on the present data, we cannot conclusively determine whether the large number of prescriptions for hearing technology in children at preschool age are interventions for acquired hearing loss at that age or for undiagnosed congenital hearing loss. Some children with hearing loss might be identified through improved tracking in the UNHS. It would be interesting to compare data from federal states with particularly comprehensive and incomprehensive tracking structures. Other studies suggest that more children with hearing loss could be detected earlier with a second screening following the UNHS [5, 15]. Therefore, a second screening between the ages of 1 and 3 would be reasonable. More studies are needed to determine the best timing for a second screening, balancing the earliest possible diagnosis with the inclusion of children with acquired hearing loss. The best method for screening needs to be discussed as well. It is necessary to compare the costs and benefits of different methods in terms of their feasibility and sensitivity.

### Interventions with different hearing technologies

In comparing the provision of hearing aids and cochlear implants, it is evident that hearing aids are the dominant type of intervention for permanent childhood hearing loss. This can be explained by the different areas of application and indication criteria. Even though cochlear implantation for children is of great importance because of the effects of profound hearing loss and the necessary interventions after surgery, this is a smaller group. Given the large number of children with hearing aids, it becomes clear that care and research for this group must not be neglected.

A prescription for hearing devices within the observation period of 1 year was documented for less than half of the children with hearing loss in the sample. If there was a gap of more than 1 year between the documentation of the diagnosis and the prescription, or if the prescription occurred prior to the diagnosis, the case fell outside the observation period. Typically, children receive a new prescription for hearing aids at intervals of 5–6 years in Germany. The high percentage of annual prescriptions of more than 40% could result from prescriptions for both ears and from new prescriptions because of progressive hearing loss. It would be interesting to consider a longer period in future studies in order to assess the comprehensiveness of interventions with hearing devices.

We cannot rule out that children with temporary conductive hearing loss also receive a diagnosis of H90.– (instead of or in addition to the diagnosis codes for diseases of the middle ear and mastoid, H65–H75). To exclude cases with temporary hearing loss, this study only considered cases where the H90 diagnosis was found as the main inpatient diagnosis or as a confirmed outpatient diagnosis in at least two of eight quarters. Excluding cases that additionally had a diagnosis for a middle ear disease did not seem sensible, as children with permanent hearing loss can indeed be affected by middle ear diseases as well. An evaluation of the ICD subgroups was not possible because, for some insured individuals, different diagnoses were documented, which would have required further validation for analysis. We have to consider that the chosen case definition may have included individuals with temporary hearing loss for which intervention with hearing technology is not necessary. Nevertheless, it is possible that among the many cases of children with a diagnosis of hearing loss and without a prescription for hearing technology in the observed period, there is a gap in healthcare provision. Future studies could provide further evidence of potential gaps in healthcare by analyzing a longer observation period. In our dataset, for 55.3% of the children in the cohort, there was no documentation of a diagnosis of hearing loss in the same quarter or in the three quarters preceding their first prescription for hearing technology. It cannot be plausibly justified that children without hearing loss would receive hearing technology, suggesting the documentation of diagnoses is incomplete.

The proportion of children with hearing technology based on claims data from statutory health insurance providers is significantly lower compared to the data from the evaluation of the UNHS [15]. However, due to the different data bases, a comparison is only possible to a limited extent. In the evaluation of the UNHS, pediatric audiology facilities were inquired about interventions for children with diagnosed bilateral congenital hearing loss to determine the provision of hearing technology [15]. The observation period for interventions was not limited to 1 year after diagnosis. The population of children with permanent hearing loss in the present study is likely larger than in the UNHS evaluation because we included children with unilateral hearing loss and any grade of hearing loss. Our data probably include more children with mild and unilateral hearing loss, which are often diagnosed later and also referred to hearing care and management later or not at all [6, 16]. Nevertheless, according to guidelines these children should also be provided with hearing technology, as they can benefit from them as well [1, 25].

### Decline in diagnoses in 2020

The estimated prevalence of hearing loss among children and adolescents in 2020 was remarkably low at 0.41% [23]. Additionally, the percentage of prescriptions for hearing devices and cochlear implantations increased despite stable or decreasing absolute numbers. It is likely that in 2020, a year characterized by COVID-19-related measures such as lockdowns and closed schools and daycare centers, many cases of hearing loss were not diagnosed due to a decrease in the use of healthcare services, rendering the estimated prevalence unreliable [11]. The delayed diagnoses may especially concern less noticeable hearing losses, such as mild or unilateral hearing loss.

As no data were available after 2020 at the time this study was conducted, it is not possible to determine whether children with hearing loss who were not diagnosed in that year will appear as delayed first diagnoses in subsequent years. However, our data suggest that the COVID-19 pandemic had negative effects on the early diagnosis and management of hearing loss for children.

### Limitations

Claims data of statutory health insurance providers offer opportunities to analyze questions about prevalence and healthcare situations without biases (self-selection, dropouts, recall, or interview bias; [18, 22]). The strengths of the data are their scope, long observation periods, and that they are population-based. There are no ethical concerns for data collection. Due to the high case numbers, claims data can be used exceptionally well for analyzing rare diseases—such as hearing loss [20]. Compared to prospective data analysis, secondary data analysis saves resources in terms of costs and time [20].

A disadvantage of secondary data analysis is that information required to answer the research question cannot be collected prospectively. Therefore, some desired information may be missing or not ideally operationalized [20]. In the present study, ICD-10 coding presented a limitation because it does not allow for differentiation by type of hearing loss (congenital or acquired) or by grade of hearing loss. Additionally, the data do not reveal the time when the hearing loss first occurred, making it impossible to determine the time interval between the onset of hearing loss and the diagnosis or prescription of hearing technology.

Uncertainties in the coding must be expected, some of which were reflected here in implausibilities. In the dataset, the code “H91—Other hearing loss” was frequently observed. Many prescribed hearing devices were not coded with a (complete) number from the medical aids index: Because the first two digits were “13,” they were assigned to the group of hearing devices but could not be assigned to a specific category of devices. At this point, it becomes clear that precise coding is important for obtaining a complete data base that can be used for research. Additionally, this study only considered data from a single health insurance provider, which imposes limitations on the generalizability of the data, as the particularities of the insured population (e.g., social status) compared to the general population can lead to bias [20].

## Practical conclusion

- In the 2010 birth cohort, 1 year after the introduction of universal newborn hearing screening (UNHS) in Germany, most children with hearing loss were provided with hearing technology after the first year of life.
- Comprehensive tracking after UNHS and additional hearing tests in early childhood are required to ensure early intervention with hearing technology for children with acquired or initially undetected hearing loss.
- Claims data provide the opportunity to analyze unresolved research questions on the prevalence of hearing loss and on the onset of hearing loss management without bias.

## Data Availability

The data were made available specifically for this project and are not publicly available. Data requests should be addressed to the data holder.

## Declarations

The use of data is regulated by the German Social Code and the supervisory authorities under data protection law. The research did not involve human participants.

The supplement containing this article is not sponsored by industry.
